# Commentary: Management of High-Grade Penile Curvature Associated with Hypospadias in Children

**DOI:** 10.3389/fped.2017.00261

**Published:** 2017-12-07

**Authors:** Smail Acimi

**Affiliations:** ^1^Visceral Surgery, Children’s Hospital Canastel, University of Oran, Oran, Algeria

**Keywords:** aetiology of hypospadias, chordee, curvature, hypospadias, pathogenesis of chordee, pathogenesis of hypospadias, proximal hypospadias

I congratulate the authors for this remarkable paper. However, the first use of profile pictures taken during successive saline erection tests to accurately measure of the correction of the penile curvature was made in 1996: the first test in the start of the operation, the second test after release of the skin and dartos fascia, the third test after possible mobilization of the urethral plate and resection of the underlying fibrous tissue, and the last test at the end of the operation (1).

Since the work published by Mettauer (2), in which he incriminated the urethral plate in the formation of chordee, the urethral plate has been systematically resected in the correction of the curvature. However, in the 1970s, King and Marshall showed that the resection of the urethral plate in curvature correction was unnecessary. Koyanagi (3), then Mollard (4) proposed the release of the chordee by mobilization of the urethral plate with resection of the underlying fibrous tissue. For Mollard, the fibrous tissue present under the urethral mucosa well delineated laterally by two large, vascular and fibrous pillars is the essential factor in the development of chordee.

We conducted a scientific study on a large number of patients to know the causes of curvature associated with proximal hypospadias; this study that lasted more than 20 years and whose results were published successively (1, 5, 6) clearly shows the essential factor responsible for the formation of the curvature associated with proximal hypospadias is the fibrosis tissue present on the ventral side of the penis. The release of the skin and dartos fascia continued widely upstream of the meatus gives a significant correction (ranging from 20 to 100°). While the mobilization of the urethral plate with resection of the underlying fibrous tissue gives a very low correction (0–20°). However, when the initial curvature is more than 90°, a short urethral plate becomes the main cause of this curvature. The curvatures by corporeal disproportion are rare and give a slight curvature.

I agree with the authors who report “It is believed that a curvature greater than 20–25° found in children is significant” (7) and I believe that a curvature <15–20° can be tolerated in the correction of the curvature. However, the curvature of the penis is an anomaly very often present in the proximal forms of hypospadias. In a recent study (182 patients) (6), the saline erection test performed at the beginning of the operation showed that only 14% of patients had a curvature (≤45°), 54% (45–90°), and 31% (≥90°). This is completely different from the findings reported by Snodgrass and the authors who report “an absence or mild curvature (<30°) in 50% of patients with proximal shaft to perineal hypospadias” (7).

One of the two main reasons for dissatisfaction of penile appearance at the adult age of patients who underwent hypospadias surgery in childhood was residual or recurrent curvature of the penis (8) and I think that in the future the real challenge for pediatric urologists will be the management of curvature associated with proximal hypospadias. For more than 20 years, we have been using a dorsal plication technique by excision of a diamond shape at the point of the maximum bend dorsally after complete separation of the dorsal neurovascular bundle from the corpus cavernosum (1, 5, 6). A procedure of dorsal plicature that seems more physiologic than others. However, it remains a difficult gesture, which requires experienced hands and especially, this gesture, was it really useful?

When the preservation of urethral plate is possible; the surgical repair of proximal hypospadias by onlay island flap or tubulization of urethral plate in the manner of Thiersch–Duplay becomes simpler, more facile, and gives excellent results. However, a significant number of adults and adolescents who we continue to follow and who have benefited during their young age an onlay island flap urethroplasty with or without mobilization of urethral plate and dorsal plication report the presence of curvature during erection (6). Thus, it is clear that there are cases where it is impossible to preserve the urethral plate in the curvature correction and it is difficult to say that the treatment in one stage of severe forms of this urogenital malformation, especially when the urethral plate was resected, is better than that realized in the two stages.

## Author Contributions

The author confirms being the sole contributor of this work and approved it for publication.

## Conflict of Interest Statement

The author declares that the research was conducted in the absence of any commercial or financial relationships that could be construed as a potential conflict of interest.
